# The Effects of Exercise Training on Recovery of Biochemical and Hematological Outcomes in Patients Surviving COVID-19: A Randomized Controlled Assessor-Blinded Trial

**DOI:** 10.1186/s40798-022-00546-4

**Published:** 2022-12-23

**Authors:** Bakhtyar Tartibian, Sirwan Mohammad Amini Khayat, Behzad Hajizadeh Maleki, Mohammad Chehrazi

**Affiliations:** 1 Department of Exercise Physiology, Faculty of Physical Education and Sport Sciences, Allameh Tabataba’i University, Tehran, Iran; 2 Department of Exercise Physiology, Faculty of Physical Education and Sport Science, Urmia University, Urmia, Iran; 3 Department of Exercise Physiology and Sports Therapy, Justus-Liebig-University, Kugelberg 62, Giessen, Germany; 4 Department of Biostatistics and Epidemiology School of Public Health, Babol University of Medical Sciences, Babol, Iran

**Keywords:** COVID-19, Exercise intervention, Randomized controlled trial, Rehabilitation

## Abstract

**Background:**

No previous research has investigated the direct effects of exercise interventions on COVID-19 outcomes. The aim is to investigate the effect of 8 weeks of home-based moderate-intensity continuous training (MICT), resistance training (RT), and combined aerobic and resistance training (CET) on biochemical and hematologic markers associated with COVID-19 symptoms and severity in COVID-19 survivors.

**Methods:**

A total of 547 male and female COVID-19 survivors were screened, and 296 (aged 20–93 years) were randomly assigned in a 1:1:1:1 ratio to one of four groups: MICT (*n* = 74), RT (*n* = 74), CET (*n* = 74), or non-exercise (NON-EX, *n* = 74). Blood samples were taken at baseline, at week 4, and week 8 after training.

**Results:**

After the intervention, compared with the NON-EX group, all 3 MICT, RT, and CET interventions caused significant improvements in the levels of creatine kinase (CK), lactate dehydrogenase (LDH), C-reactive protein (CRP), troponin-I, d-dimer, creatinine, urea, potassium (K), sodium (Na), white blood cell (WBC), neutrophils, lymphocytes, red blood cells (RBC), platelets, hemoglobin, and hematocrit concentrations (*P* < 0.05). CET was effectively superior to MICT and RT in the improvements in the biochemical and hematological variables studied (*P* < 0.05).

**Conclusions:**

Overall, the present study demonstrates that long-term MICT plus RT interventions have a synergistic effect in accelerating and enhancing the recovery in patients surviving COVID-19.

*Trial registration* IRCT20160605028270N3, 6 September 2020.

**Supplementary Information:**

The online version contains supplementary material available at 10.1186/s40798-022-00546-4.

## Key Points

- No prior studies have investigated the direct impact of exercise interventions on COVID-19 outcomes.
- Regular exercise training accelerates and improves recovery in COVID-19 survivors.
- Eight weeks of aerobic training, resistance training, or combined aerobic and resistance training improved considerably the biochemical and hematologic indicators associated with COVID-19 symptoms and severity.
- Combined aerobic and resistance training was substantially more effective in improving these biomarkers and indicators than aerobic training alone and resistance training alone.

## Introduction

The number of cases of infected persons has been rising dramatically since the new coronavirus (SARS-CoV-2) outbreak in December 2019, resulting in the coronavirus illness (COVID-19) being designated a worldwide pandemic on March 11, 2020. Globally, there have been more than 237 million cases and 4.8 million fatalities as of October 10, 2021. The Clinical characteristics of the disease are quite varied, and the severity of the condition can range from asymptomatic to lethal. In addition to respiratory symptoms, evidence of COVID-19 attacks to several organs such as digestive, urinary, cardiovascular systems, the spleen, the lymph nodes as well as neurological and neuromuscular impairments has been reported. Moreover, some hematological and biochemical markers, including white blood cell (WBC), lymphopenia, platelet, C-reactive protein (CRP), lactate dehydrogenase (LDH), creatine kinase (CK), d-dimer, creatinine, and troponin, are used as prognostic markers in COVID-19 and increased circulatory levels of these markers and indicators have already been linked to disease severity [1–4]. Mounting evidence, from studies that have mostly focused on COVID-19 patients following hospitalization, also shows that a significant number of patients do not fully recover and seem to have long-term consequences, described by a variety of different terminologies that aren't all agreed on, including chronic COVID-19, post-COVID-19 syndrome, post-acute (or late) COVID-19 complications, and post-acute COVID-19 syndrome [5–8]. Furthermore, one research with limited sample size and no comparison group of individuals without COVID-19 documented the long-term consequences of COVID-19 patients who did not require hospitalization [9].

It is generally confirmed that regular physical activity enhances immune function and that active individuals have a reduced incidence, severity of symptoms, and morbidity from viral infections [10–12]. Regular physical activity lowers the risk of systemic inflammation, improves cardiorespiratory health, and strengthens muscles, the mechanisms through which it alleviates the symptoms of various acute and chronic health conditions [13, 14]. One of the latest topics to be investigated in this field is the association between physical activity and COVID-19 outcomes [11, 12, 15, 16]. The literature review shows that a higher degree of physical activity is linked to a decreased risk of COVID-19-related hospitalizations [17]. Also, a recent study by Sallis et al. concluded that physical inactivity is linked to a greater incidence of COVID-19 complications [18]. However, a key problem with these studies is that they have simply relied on observational self-reported data on physical activity, and, as far as we know, no previous research has investigated the direct effects of exercise interventions on COVID-19 outcomes.

Considering the literature as mentioned above, therefore, we conducted a randomized controlled trial to investigate the effects of 8 weeks of home-based moderate-intensity continuous training (MICT), resistance training (RT), and combined aerobic and resistance training (CET) on biochemical and hematologic markers linked to COVID-19 symptoms and severity in surviving patients infected with the disease. To our knowledge, this is the first study to address this issue in COVID-19 patients.

## Methods

### Design

This randomized controlled assessor-blinded trial assessed the effect of 8 weeks of home-based MICT, RT, and CET on biochemical and hematologic markers associated with COVID-19 symptoms and severity in COVID-19 survivors. The protocol is available on the trial website (www.irct.ir—IRCT20160605028270N3). The Ethics Committee of Allameh Tabataba'i University in Tehran, Iran, approved the trial (Reference Number: IR.ATU.REC.1399.061), which was conducted in accordance with the Declaration of Helsinki. Written informed consent was obtained from all patients before enrolment.

### Patients

The main entry criterion was laboratory confirmation of COVID-19. Additional requirements were (1) sedentary lifestyle and physical inactivity for at least 6 months; (2) no history of use of cigarette and alcohol in the last 6 months; (3) no history of following a regular diet over the previous 12 months; (4) ability to participate and raise their level of physical activity; and eligibility after respiratory, cardiovascular, endocrine, and metabolic evaluations.

Male and female (aged 20–93 years) patients with laboratory-confirmed COVID-19 who were hospitalized in Masih Daneshvari, Sina and Atieh Hospitals, Tehran, Tehran Province, Iran, between June 15, 2020, and September 27, 2020, were identified. Based on computed tomography (CT) results and a positive result for SARS-CoV-2 in a qRT-PCR test of nasal and pharyngeal swab specimens following WHO standards, laboratory confirmation for COVID-19 was accomplished [19]. The 7th edition of China's National Health Commission's New Coronavirus Pneumonia Prevention and Control Program was used to assess the diagnosis and severity of COVID-19.

Patients were initially selected and given a study information booklet by nurse managers on the wards as a third party before the trial began. Patients (*n* = 547) who agreed to take part in this study signed a written informed consent form, exchanged contact information, and were instructed to notify the research team before leaving the hospital. Consequently, within 24 h after being discharged from the hospital, all the patients were approached by a trained research assistant. They were given the necessary information in both written and spoken form, and investigators recorded their biographical data and physical characteristics.

It needs to be mentioned that the primary research protocol registered on the trial registry is a pervasive project and includes samplings of different tissues at time points of baseline, 4, 8, 12, and 16 weeks and one month of de-training in COVID-19 survivors. However, in the present study, due to the importance and attractiveness of the topic, we decided to probe prognostic hematological and biochemical markers within the blood samples in a more limited population and timetable than the original design. Accordingly, the participants of the present study were randomly selected from the subjects of the main design to examine the response of prognostic blood markers of the disease to different patterns of physical activity in patients over eight weeks.

### Sample Size

Our preliminary results (a pilot study) on physical activity and COVID-19 outcomes, which demonstrated an effect size (Cohen d) of 0.41, and expert opinion, were used to determine sample size. An attrition rate of 15% was estimated. Recruitment of 296 individuals (74 participants in each group) was required to provide a 90% statistical power (*α* = 0.05) to identify a significant effect of different home-based exercise interventions over NON-EX controls. The initial sample size estimated to achieve 90% statistical power was 252, and after adding up 15% attrition we reached 296 participants. 296 − 0.15 * 296 = 85 * 296/100 = 252.

### Randomization and Blinding

**Fig. 1:**
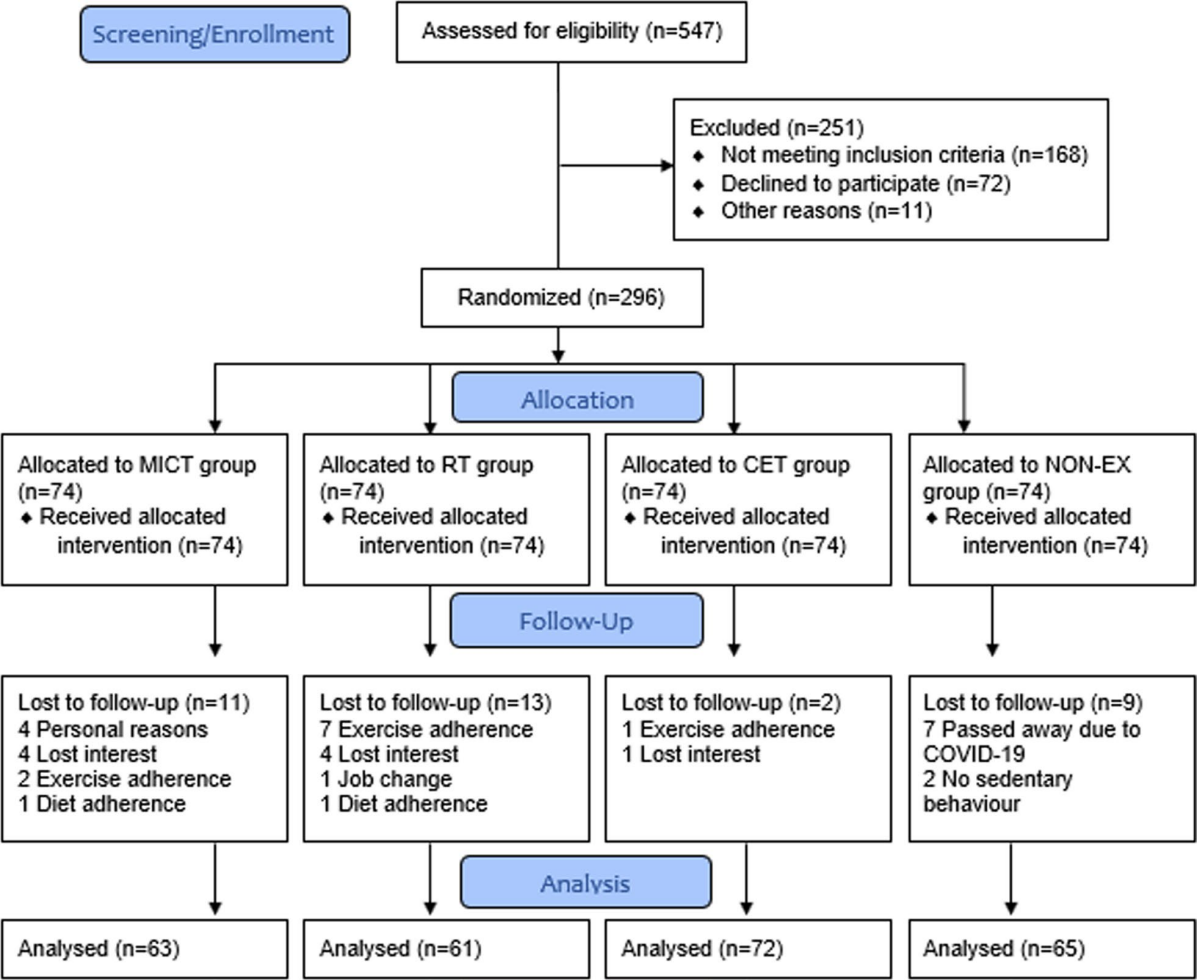
Follow-up diagram. MICT: moderate intensity continuous training; RT: resistance training; CET: combined exercise training; NON-EX: non-exercise

In a single research facility in Iran, 296 patients who met the inclusion criteria signed a written informed consent form and were randomized in a 1:1:1:1 ratio to one of four groups: MICT (*n* = 74), RT (*n* = 74), CET (*n* = 74), and non-exercise (NON-EX, *n* = 74) (Table 1, Fig. 1). Block randomization was done by a computer-generated random allocation sequence with random block sizes (4–8), and group assignments were kept in a sealed opaque envelope that was opened by the research coordinator at randomization. The sequence was randomly generated by the website www.sealedenvelope.com. Participants, exercise specialists, and physicians were not blinded, whereas assessors were masked to randomization probabilities.

**Table 1:** Demographic and clinical characteristics of patients and participants at baseline

Characteristics	MICT (*n* = 63)	RT (*n* = 61)	CET (*n* = 72)	NON-EX (*n* = 65)
Sex (male/female)	45/18	49/12	55/17	46/19
Age (years), Mean ± SD	55.0 ± 29.1	56.2 ± 26.9	59.6 ± 34.3	59.4 ± 36.5
Age category, *N* (%)				
20–30 years	11 (17.5)	10 (16.4)	11 (15.3)	10 (15.4)
31–40 years	11 (17.5)	11 (18.0)	12 (16.7)	10 (15.4)
41–50 years	8 (12.7)	8 (13.1)	9 (12.5)	9 (13.8)
51–60 years	8 (12.7)	7 (11.5)	9 (12.5)	9 (13.8)
61–70 years	8 (12.7)	8 (13.1)	10 (13.9)	10 (15.4)
71–80 years	8 (12.7)	8 (13.1)	10 (13.9)	8 (12.3)
81–90 years	5 (7.9)	5 (8.2)	7 (9.7)	5 (7.7)
91–100 years	4 (6.3)	4 (6.6)	4 (5.5)	4 (6.2)
Severity, *N* (%)				
Mild	6 (9.5)	5 (8.2)	7 (9.7)	6 (9.2)
Moderate	10 (15.9)	13 (21.3)	12 (16.7)	8 (12.3)
Severe	25 (39.7)	23 (37.7)	28 (38.9)	30 (46.2)
Critical	22 (34.9)	20 (32.8)	25 (34.7)	21 (32.3)
Symptoms, *N* (%)				
Fever	54 (85.7)	51 (83.6)	54 (75.0)	52 (80.0)
Cough	34 (54.0)	38 (62.3)	37 (51.4)	32 (49.2)
Sore throat	9 (14.3)	11 (18.0)	13 (18.0)	8 (12.3)
Myalgia	21 (33.3)	21 (34.4)	24 (33.3)	22 (33.8)
Dyspnoea	27 (42.9)	26 (42.6)	23 (31.9)	30 (46.2)
Chest pain	34 (54.0)	37 (60.7)	34 (47.2)	40 (61.5)
Diarrhea	11 (17.5)	9 (14.8)	12 (16.7)	9 (13.8)
Time between diagnosis and the first sampling (day), Mean ± SD	9.7 ± 2.3	10.2 ± 1.5	10.0 ± 2.5	9.32 ± 4.6
Weight (kg), Mean (SD)	72.0 ± 7.2	74.7 ± 13.7	71.6 ± 11.0	72.1 ± 9.9
Fat (%)	27.6 ± 3.6	29.4 ± 4.1	28.6 ± 3.3	28.8 ± 3.7
Body mass index (kg/m^2^), Mean (SD)	25.8 ± 2.3	27.4 ± 4.3	26.9 ± 2.9	26.3 ± 2.5
Body mass index category, *N* (%)				
< 25 kg/m^2^	23 (36.5)	11 (18.0)	17 (23.6)	23 (35.4)
25–29.99 kg/m^2^	38 (60.3)	41 (67.2)	48 (66.7)	37 (56.9)
≥ 30 kg/m^2^	2 (3.2)	9 (14.8)	7 (9.7)	5 (7.7)
Waist circumference (cm)	94.2 ± 11.4	99.1 ± 11.4	96.2 ± 11.6	96.2 ± 10.9
Hip circumference (cm)	103.6 ± 6.6	107.0 ± 9.0	104.1 ± 7.1	104.8 ± 8.8
DEC (kcal)	1851.2 ± 195.1	1973.1 ± 314.3	1853.0 ± 262.6	1834.9 ± 257.0
MET (kcal/kg/hour)	1.07 ± 0.06	1.08 ± 0.07	1.06 ± 0.06	1.06 ± 0.06
Marital status, *N* (%)				
Married/living with partner	31 (49.2)	31 (50.8)	33 (45.8)	29 (44.6)
Divorced/separated	7 (11.1)	5 (8.2)	9 (12.5)	10 (15.4)
Widowed	14 (22.2)	15 (24.6)	18 (25.0)	16 (24.6)
Never married	11 (17.5)	10 (16.4)	12 (16.7)	10 (15.4)
Number of children, Mean ± SD	2.3 ± 1.3	2.1 ± 0.9	3.2 ± 0.5	2.9 ± 1.7
SpO_2_, *Mean* ± *SD*	89.4 ± 1.0	89.1 ± 0.8	88.5 ± 1.3	88.5 ± 3.1
Corticosteroid therapy, *N* (%)	29 (46.0)	20 (32.8)	27 (37.5)	31 (47.7)
Antiviral therapy, *N* (%)	34 (54.0)	41(67.2)	45 (62.5)	34 (52.3)

*MICT* moderate intensity continuous training, *RT* resistance training, *CET* combined exercise training, *NON-EX* non-exercise, *DEC* daily energy cost, *MET* metabolic equivalent

### Procedures

#### Home-Based Exercise Interventions

Patients in the intervention groups took part in a progressive home-based program of MICT, RT, and CET regimen three times a week for eight weeks. They were also given customized instruction and a demonstration on how to use the heart rate (HR) monitor to monitor and record HR, how to use the 6- to 20-point Borg RPE Scale [20], and how to report any problems they had during the walking or resistance training workouts. A study team member (exercise specialist or nurse) supervised weekly home-based sessions for four consecutive weeks and then every other week. The exercise prescription was modified at each home visit, gradually increasing both walking and resistance exercise. Patients in the exercise group were also given a DVD and an exercise manual to use during their unsupervised sessions as a reference [21]. During the intervention phase, the patients were asked to maintain their usual daily routines and not change their lifestyles other than to comply with the study's requirements.

#### MICT Protocol

The MICT protocol consisted of progressive low- to moderate-intensity walking. We used the HR reserve technique [22] to determine intensity level based on the Six-Minute Walk Test, with patients starting at 50% intensity and advancing to 65% intensity for a minimum of 15 min and a maximum of 40 min three to five times per week. Patients were told to keep RPE between 12 and 14 [20]. Only the first week of walking was supervised to guarantee patient safety and understanding of self-monitoring procedures (RPE, HR, blood pressure). Patients walked unsupervised and conducted self-monitoring autonomously for the remaining of the intervention period if there were no safety problems following the first session. During weekly home visits, however, activity records (step/cord calendars) were checked, and walking prescriptions were modified as needed [21].

#### RT Protocol

The RT protocol was done with color-coded Thera-cords^d^ in which the cord's hue reflects various levels of resistance. To acquaint themselves with the training regimen and for safety concerns, all the patients started with a yellow or red cord, which represents the lowest resistance level. The resistance exercise sessions lasted about 15–40 min, depending on the patient's tolerance, and included a 5-min warm-up (low-intensity flexibility/stretching) followed by a 15- to 40-min lower- and upper-body resistance workout. Ankle plantarflexion and dorsiflexion, hip extension, abduction, and adduction, knee extension and flexion, and leg extension were among the lower-body resistance exercises. Workouts for wrist extension and flexion, elbow extension and flexion, and shoulder abduction, flexion, and rotation were included in upper-body resistance training. When the patients completed two sets of 12–15 repetitions or scored less than 14 on the RPE scale, their resistance training progress was tracked and modified. Weekly supervised sessions were utilized to track how the resistance training regimen was working. During weekly home visits, however, activity records (cord calendars) were checked, and resistance prescriptions were modified as needed [21]. To minimize muscular fatigue and soreness, patients were instructed to practice the resistance exercises 3 times each week, but not on sequential days. A seated regimen using the identical exercise routines devised by the investigators was employed by some patients who could not undertake standing activities. The majority of patients advanced to 3 sets of 15 repetitions 3 times weekly over the 8-week trial period [21].

#### CET Protocol

The main program consisted of MICT followed by the RT and lasted about a minimum of 15 min and a maximum of 40 min. The training progression was similar to that illustrated in the MICT and RT program sections.

#### Exercise Adherence

Step/cord calendars and pedometers were used to measure exercise adherence. The Omron HJ 112 was used to estimate the number of daily steps taken. We evaluated steps reported by patients on the step/cord calendar with actual pedometer data to confirm patient documentation because the device resets at midnight automatically. Thera-cord color, exercises done, and the number of repetitions completed on the step/cord calendar were used to track adherence to the resistance exercise component. Using the step/cord calendar, the nurse/exercise specialist documented adherence from the past week on the intervention log page for each patient each week. Three recorded walking and strength-training sessions per week were necessary to be considered 100 percent adherent to the exercise program [21].

#### Exercise Progression

For both aerobic and resistance training, the major technique of progressing exercise was based on RPEs. During the aerobic exercise, patients were instructed to record their maximum heart rate, RPE, the number of steps taken, or symptoms on a step/cord calendar. Patients were also instructed to keep track of their Thera-cord color and number of repetitions after each session, as well as their maximal HR, RPE, and any symptoms they had. For all forms of exercise, patients were told to keep their target HR within a defined range and their RPE below 15 [11].

### Samplings

At baseline (24 h after hospital discharge), at the end of week 4 and 8, all patients were transported to the lab (between 0700 and 0800), and 10 ml blood samples were drawn. Patients were fasting (except for water) for 12 h and free of exercise at least 24 h. in advance of the sampling. Serum was collected and preserved at − 80 °C to analyze within a week. Both groups had a similar number of visitations during the intervention.

### Outcomes and Measurements

#### Primary Outcomes

Primary outcomes were CRP, troponin-I, d-dimer. RANDOX (UK) CRP full Range kit with an assay range of 0.18–165 mg/L was used to determine CRP levels by using Latex-Enhanced Immunoturbidimetric Assay in Roche Hitachi 917 biochemistry analyzer (Switzerland). Atellica IM High-Sensitivity Troponin I Assay Analyzer device (SIEMENS, Germany) by dual-capture sandwich immunoassay methos was used to determine Troponin-I concentrations. The limit of blank (LoB) for the Atellica IM TnIH Assay was established as 0.50 ng/L (pg/mL) and the limit of detection (LoD) as 1.60 ng/L (pg/mL). D-dimer levels (measuring range, 0.1–4.0 μg/mL) were measured by Cobas h 232 POC system (Switzerland).

#### Secondary Outcomes

Secondary outcomes were CK, LDH, creatinine, urea, K, and Na concentrations. CK, LDH, creatinine, and urea concentrations were determined spectrophotometrically by using Pars Azmun (Iran) kit in an auto-analyzer device (BT3000, Italy). The intra-assay and interassay coefficients of variation (CVs) for the CK assay were 2.00% and 2.12%, respectively. The intra-assay and interassay CVs for the LDH assay were 3.86% and 2.13%, respectively. The intra-assay and interassay CVs for the creatinine assay were 6.45% and 3.63%, respectively. The intra-assay and interassay CVs for the urea assay were 5.41% and 5.79%, respectively. Na (measuring range, 80.0–200.0 mmol/L) and K (measuring range, 0.50–15.00 mmol/L) concentrations were measured by Convergys® ISE Full- automatic electrolyte analyzer (Germany) based on the advanced ion selective electrode (ISE) technology. Hematological parameters including WBC, neutrophils, lymphocytes, RBC, platelets, hemoglobin, and hematocrit also were analyzed using Sysmex KX-21N hematology analyzer (Sysmex Corporation Kobe, Japan).

#### Dietary and Medication Intake Measures

Qualified dietitians used a standardized semi-quantitative food frequency questionnaire (FFQ) to gather dietary data at baseline and every week after the baseline assessment. During the trial, subjects were expected to continue their regular diet and were advised to consume a diet that was as comparable as feasible on each sampling day. Standard and self-reported questionnaires were also used to collect data on the usage of medicines and supplements.

#### Self-Reported Sleep Quality

The Pittsburgh Sleep Quality Index [PSQI] is a commonly used questionnaire that analyzes subjective sleep quality as well as quantitative sleep–wake characteristics (measures of sleep latency, duration, and efficiency obtained from self-report) during the previous month. The PSQI's 19 questions are scaled into seven component scores, which are added together to provide a global PSQI score ranging from 0 to 21, with higher scores indicating worse sleep [23]. Participants were asked to fill PSQI out at the end of each month of intervention.

### Missing Data

Thirty-five patients (MICT, *n* = 11; RT, *n* = 13; CET, *n* = 2; and NON-EX, *n* = 9) could not complete the study protocol and accordingly were excluded from the study. Consequently, 261 patients remained in the analysis. The reasons for exclusion are provided in Fig. 1.

### Statistical Analysis

For continuous and categorical variables, data are presented as mean ± SD or frequency (percentage). Demographics and baseline characteristics were compared across intervention groups using one-way analysis of variance (ANOVA) with a post hoc test of either Bonferroni (in case of homogeneity of variances) or Dunnett T3 (in case of heterogeneity of variances). Split-plot repeated measure ANOVA was used to compare mean continuous variables as the main effect across the four intervention groups and to assess time trend responses and its interaction by the intervention while controlling for baseline measurement. Bonferroni adjustment was used as the post hoc test. We used Mauchly's test of sphericity as a fundamental assumption for repeated measures ANOVA. If the assumption was violated, a Greenhouse–Geisser correction was applied. Eta squared effect sizes presented in Additional file 1: Table S1 were estimated by the repeated measures ANOVA model. A rule of thumb is usually applied to interpret eta squatted. An effect size of 0.01 is considered a small one, while an eta squared around 0.06 and 0.14 or higher was interpreted as medium and high effect size, respectively. STATA version 15 was used for all statistical analysis and computations. GraphPad Prism version 8 was used to create the figures. A statistical test was considered significant if *P* value < 0.05.

## Results

In the factors investigated, there were no significant differences in baseline characteristics between the four research groups (*P* > 0.05).

### Dietary and Medication Intake

The food and medication intakes of the patients were comparable across all groups, and there were no significant differences in dietary and medication intakes between or within groups during the course of the study (*P* > 0.05).

### Self-Reported Sleep Quality

There was no significant difference between groups in self-reported sleep quality during the study (*P* > 0.05).

### Biochemical Markers

**Fig. 2:**
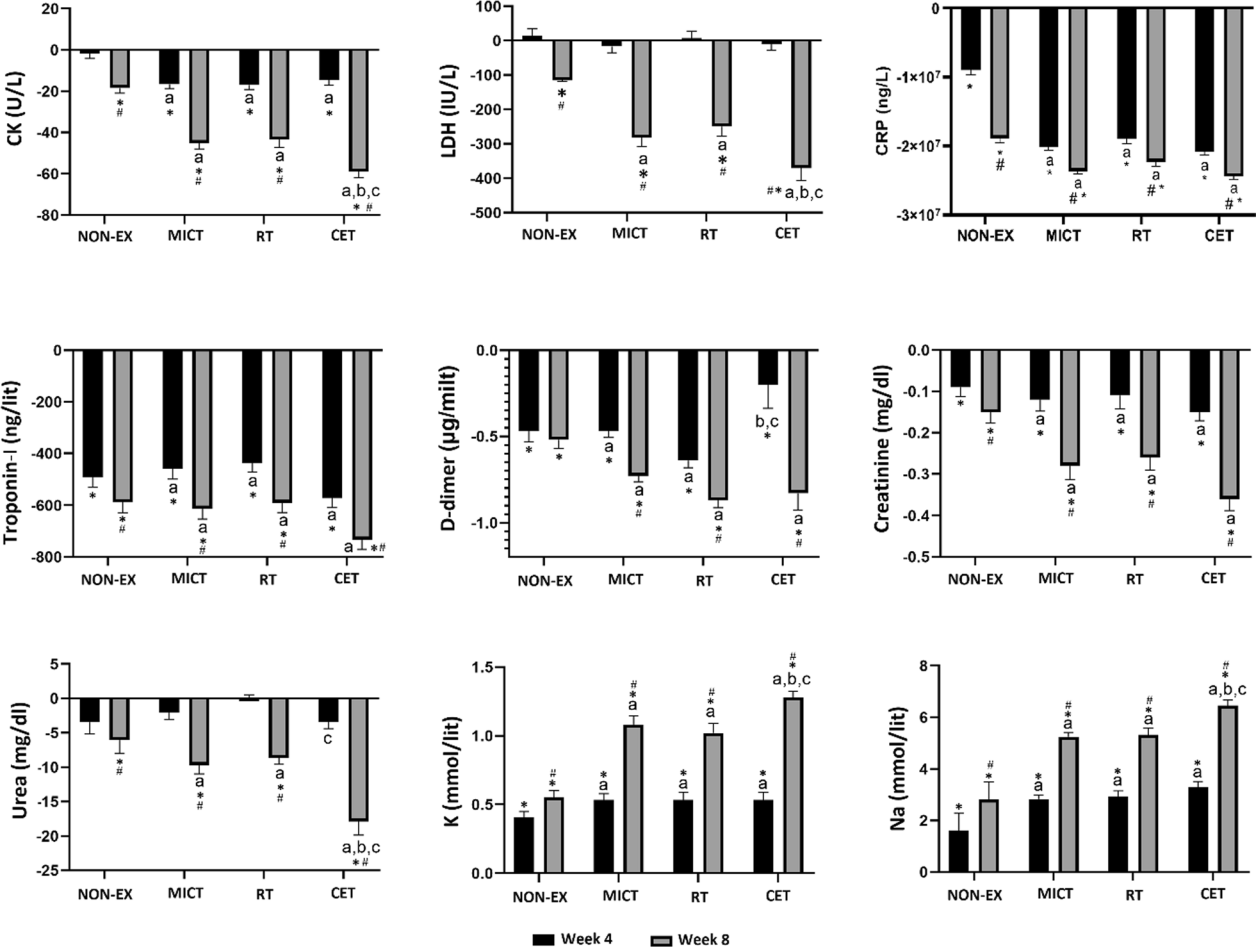
Changes from baseline in biochemical markers in the four groups of surviving patients infected with COVID-19 at weeks 4 and 8. MICT: moderate intensity continuous training; RT: resistance training; CET: combined exercise training; NON-EX: non-exercise. *P < 0.05, significant changes from baseline. #*P* < 0.05, significantly different from week 4 values (within groups, week 4 vs. week 8). Superscripts denote significant differences among the groups (NON-EX = a; MICT = b; RT = c; and *CET* = d)

Attenuated levels of CK compared to baseline were observed at weeks 4 and 8 in the MICT, RT and CET groups (*P* < 0.0001) (Fig. 2). The decrease was significantly greater in the CET group (*P* < 0.0001), and all three exercise intervention groups had a greater decrease than the NON-EX group (*P* < 0.0001). The MICT and RT acted synergistically to cause the decrease in CK. These levels improved from baseline at 8 weeks in the NON-EX group.

At 4 weeks of the intervention, LDH was not significantly different than baseline values and there were no differences between group values (Fig. 2). However, by 8 weeks, the MICT and RT acted synergistically to cause the decrease in LDH (*P* < 0.0001). By 8 weeks, there were significant improvements in LDH in the NON-EX group; however, these changes were significantly smaller (*P* = 0.002) than changes seen in the MICT, RT and CET groups (*P* < 0.0001).

Attenuated CRP, troponin-I, and creatinine levels compared to baseline were observed at weeks 4 and 8 in the MICT, RT, and CET groups (*P* < 0.0001), and there were no differences between group values (Fig. 2). Also, by 4 and 8 weeks, there were significant improvements in CRP, Troponin-I, and creatinine levels in the NON-EX group; however, these changes were significantly smaller (*P* < 0.0001) than changes seen in the MICT, RT, and CET groups (*P* < 0.0001).

At 4 and 8 weeks, d-dimer levels had decreased from baseline (*P* < 0.0001) in the MICT, RT, and CET groups (Fig. 2). In the MICT, and RT groups these levels were greater than observed in the NON-EX group (*P* < 0.0001). At 8 weeks, the CET concentrations of d-dimer were only significantly greater than the NON-EX group (P < 0.0001). This decrease was significantly lower than the MICT, and RT groups at week 4. These levels decreased at both 4 and 8 weeks in the NON-EX group (P < 0.0001).

Attenuated levels of urea compared to baseline were observed at week 8 in the MICT, RT and CET groups (*P* < 0.0001) (Fig. 2). The decrease was significantly greater in the CET group (*P* < 0.0001), and all three exercise intervention groups had a greater decrease than the NON-EX group (*P* < 0.0001). The MICT and RT acted synergistically to cause the decrease in urea. These levels improved from baseline at 8 weeks in the NON-EX group.

### Electrolytes

**Fig. 3:**
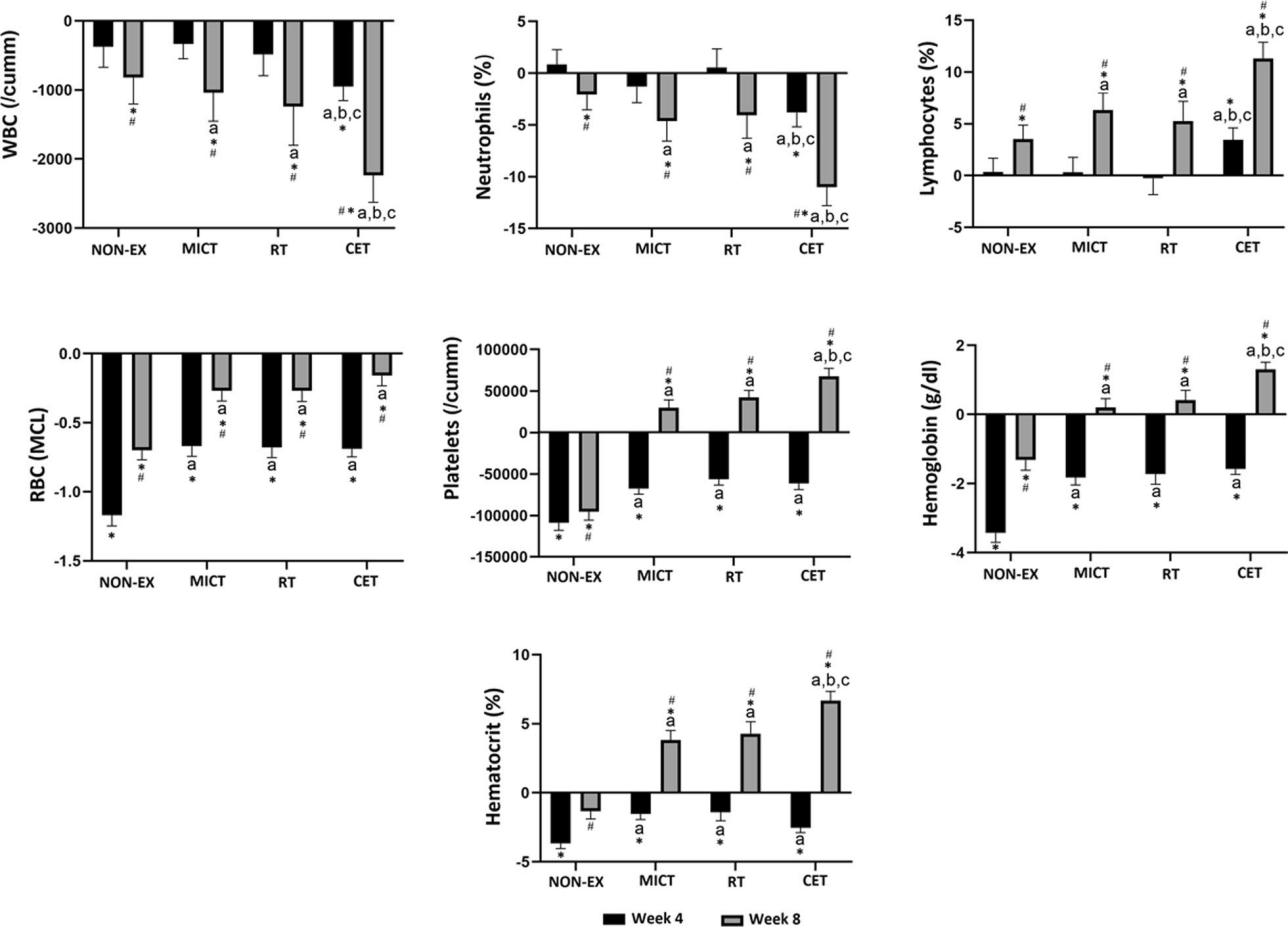
Changes from baseline in immune cell counts and hematological markers in the four groups of surviving patients infected with COVID-19 at weeks 4 and 8. MICT: moderate intensity continuous training; RT: resistance training; CET: combined exercise training; NON-EX: non-exercise. *P < 0.05, significant changes from baseline. #*P* < 0.05, significantly different from week 4 values (within groups, week 4 vs. week 8). Superscripts denote significant differences among the groups (NON-EX = a; *MICT* = b; RT = c; and *CET* = d)

At 4 and 8 weeks, K and Na levels had improved from baseline (*P* < 0.0001) in the MICT, RT, and CET groups (Fig. 3). The improvement was significantly greater in the CET group (*P* < 0.0001), and all three exercise intervention groups had a greater increase than the NON-EX group (*P* < 0.0001). The MICT and RT acted synergistically to cause the increase in K and Na. These levels improved from baseline at 4 and 8 weeks in the NON-EX group.

### Hematologic Parameters

Attenuated levels of WBC, neutrophiles, and lymphocytes compared to baseline were observed at week 8 in the MICT, and RT groups (*P* < 0.05) (Fig. 3). These levels changed at both 4 and 8 weeks in the CET group (P < 0.0001). The alterations were significantly greater in the CET group (*P* < 0.0001), and all three exercise intervention groups had a greater variation than the NON-EX group (*P* < 0.0001). The MICT and RT acted synergistically to cause the changes in WBC, neutrophiles, and lymphocytes. Also, these levels changed from baseline at 8 weeks in the NON-EX group.

At 4 and 8 weeks, RBC levels had improved from baseline (*P* < 0.0001) in the MICT, RT, and CET groups (Fig. 3). The improvement was significantly greater in the CET group (*P* < 0.05), and all three exercise intervention groups had greater improvements than the NON-EX group (*P* < 0.0001).

At 4 weeks of the intervention, platelets, hemoglobin, and hematocrit values significantly decreased than baseline in the MICT, RT, and CET groups, with no differences between group values (*P* < 0.0001) (Fig. 3). However, by 8 weeks, the MICT, RT, and CET interventions resulted in significant increases in platelets, hemoglobin, and hematocrit from the baseline value (*P* < 0.0001). The alterations in these values were significantly greater in the CET group (*P* < 0.0001). The MICT and RT acted synergistically to cause the changes in platelets, hemoglobin, and hematocrit, but it appears that the RT contributed more so to these changes. In the NON-EX group, platelets and hemoglobin levels decreased from baseline at 4 and 8 weeks. Also, hematocrit levels declined from baseline at 4 weeks in this group. The alterations in platelets, hemoglobin, and hematocrit at week 4 were significantly greater in the NON-EX group (*P* < 0.0001). Additional file 1: Table S1 shows the eta effect size computed separately for each time period.

## Discussion

### Principal Findings

The novel findings of this study are: (1) improvements in CK, LDH, CRP, troponin-I, d-dimer, Creatinine, urea, K, Na, WBC, neutrophils, lymphocytes, RBC, platelets, hemoglobin, and hematocrit were seen with eight weeks of MICT alone and with RT alone in this cohort of patients surviving COVID-19, (2) the MICT acted synergistically with the RT to induce profound alleviations in the biochemical and hematological variables studied. Overall, these results go beyond previous reports, showing that exercise training accelerates and enhances the recovery in patients surviving COVID-19.

Almost all COVID-19 patients who were hospitalized had increased serum CK and LDH values, possibly due to the involvement of virus-mediated systemic inflammation, myotoxic cytokines, or direct muscle toxicity [24]. The literature review shows that CK levels at admission are higher in COVID-19 patients who experience more severe outcomes afterward [25]. Elevated levels of LDH were also found to be associated with disease severity and mortality in these patients [26]. In this study, the serum CK and LDH levels of all three MICT, RT, and CET groups improved significantly with different kinetics for the three types of training modalities. The CET alterations of these parameters were substantially greater than those seen in the MICT and RT groups. The mechanisms behind the training-mediated improvement in serum CK and LDH levels in COVID-19 patients are unknown; however, the exercise training appears to attenuate these levels by reducing their leakage from the tissue into the serum or accelerating their clearance [27, 28], which suggests an adaptation to training program in this cohort [29–31]. These changes seem to contribute to a more positive recovery and decrease the risk of cardiovascular and renal events in COVID-19 patients, as higher levels of these enzymes have previously been linked to coagulopathy, myocardial injury, and renal failure in these patients [32].

As a marker of severe infection and systemic inflammation, CRP levels were shown to be substantially higher in the early stages of infection in severe COVID-19 patients, even before CT scans revealed serious abnormalities. CRP has been linked to disease progression and is an early indicator of severe COVID-19 [33]. In patients with COVID-19, elevated serum CRP levels are also highly linked to venous thromboembolism, acute renal damage, critical illness, and in-hospital fatality [34]. Our findings parallel previous reports that exercise training in the form of MICT, RT, and CET attenuates serum CRP levels [35–37]. Although the exact processes by which exercise training reduces CRP are unknown, numerous possible explanations exist. One pathway is through the control of interleukins and associated cytokines, such as IL-6 and TNF-α, which are both generated either by adipose tissue or by peripheral blood mononuclear cells, and the former increases hepatic CRP production. The levels of IL-6 and TNF-α were not measured in this study; however, earlier studies who employed the MICT [38], RT [39], and CET [40] protocols to examine the effect of exercise training on the levels of interleukins and associated cytokines found that exercise training had an attenuating impact on these parameters. Regarding the effect of different exercise modalities on CRP levels in COVID-19 patients, it seems that exercise training is an effective mean in inducing reduction in systemic inflammation and infection, thus possibly improving renal and coronary risk profiles as well as the clinical outcomes in this population.

In COVID-19 patients, myocardial injury as measured by an increased troponin-I is an independent predictor of mortality and other negative clinical outcomes [41]. In hospitalized COVID-19 patients, these levels had prognostic significance, and patients with greater troponin levels, in particular, were more likely to be admitted to the critical care unit, and had a higher in-hospital death rate [42, 43]. Troponin-I elevation in COVID-19 patients could result from viral myocarditis, microangiopathy, cytokine-driven myocardial damage, type II myocardial infarction, and acute coronary syndrome [44]. Our findings show that all three MICT, RT, and CET treatments significantly reduced troponin-I serum levels in COVID-19 survivors at both 4 and 8 weeks. These findings are similar to those reported by Koppen et al*.* [45], who found reductions in serum high-sensitivity cardiac troponin T concentrations in patients with heart failure with reduced ejection fraction following 12 weeks of moderate continuous training, high-intensity interval training, or a recommendation of regular exercise when adjusted for clinical variables. Higher levels of peak oxygen uptake have already been adversely associated with variations in serum high-sensitivity cardiac troponin T concentrations [45], suggesting that training-induced increases in aerobic capacity may be the driving factor underlying the post-exercise troponin response. On this basis, troponin-I elevations in COVID-19 patients may represent preclinical pathophysiological antecedents of hemodynamic stress and damage that can be prevented with regular training modalities.

Severe cases of COVID-19 have been linked to infection-mediated coagulopathy and subsequent hyperfibrinolysis [46]. Multiple investigations have found a link between the procoagulant marker d-dimer elevation [47], lymphocytopenia [48], neutrophilia [49], leukocytosis [50], and thrombocytopenia [51] with impaired coagulation function, severe disease manifestation and higher mortality in COVID-19 patients [52, 53]. We studied the variance in these levels in response to different exercise modalities to see if regular exercise interacts with the recovery of COVID-19-associated coagulopathy indicators. Surprisingly, no clinical investigation has probed these interactions in COVID-19 survivors yet; however, our research and earlier interventional studies have demonstrated that regular exercise regimens produce a positive coagulation response [54, 55]. The post-intervention improvements in the levels of COVID-19-associated coagulopathy indicators in this cohort of COVID-19 survivors may be related to the training-induced adaptations in both the coagulation and fibrinolytic systems. Thus, regular exercise training in the form of MICT, RT, and CET appears to accelerate the recovery and benefit the COVID-19 patients' coagulation and fibrinolytic system positively and may reduce cardiovascular risks in this group of surviving patients.

COVID-19 causes tubular injury in the kidneys, which is often accompanied by urinalysis abnormalities. Glomerular filtration is also impaired in COVID-19 patients, as seen by elevated blood creatinine and urea nitrogen levels [56, 57]. Electrolyte abnormalities such as hyponatremia and hypokalemia are additionally associated with the disease severity in COVID-19 patients [58]. To date, the research has been confined to observations made during hospitalization; as a result, the recovery of renal condition in COVID-19 patients who survive hospitalization is unclear. In the present investigation, we studied the recovery of renal function in response to exercise training in COVID-19 survivors. All three MICT, RT, and CET treatments resulted in substantial improvements in creatinine, urea nitrogen, sodium, and potassium from baseline values at 4 and 8 weeks, compared to the NON-EX group. Importantly, MICT and RT treatments show a synergistic impact in improving urea nitrogen, sodium, and potassium levels after eight weeks of intervention. We could not find any study investigating the impacts of aerobic, resistance, or mixed regimens on renal recovery in COVID-19 survivors. However, regarding renal function, previous research has demonstrated that frequent exercise sessions can change renal hemodynamics by increasing blood flow, which stimulates muscles and leads to an increase in intraglomerular pressure. As a result, an improvement in efferent arteriole pressures after exercise is hypothesized to increase hydraulic pressures, allowing proteins to pass through the glomerulus more easily [59, 60]. Therefore, a substantial improvement in concentrations of these renal function indicators following exercise training likely represents continued subclinical development of renal pathology in COVID-19 survivors. This observation could be associated with the enhanced recovery of renal function and the increased abilities of the kidneys to regulate electrolytes in COVID-19 survivors.

Our findings parallel previous reports that COVID-19 perturbates RBC, hemoglobin, and hematocrit concentrations [61]. In patients with severe COVID-19 infection, anemia, particularly inflammation-related anemia, is common, and this is linked to extended hospitalizations, poor clinical outcomes, and poor survival. This might be connected to decreased tissue oxygenation and anemia, reflecting comorbidities such as compromised renal function, advanced age, or increased inflammation [61]. Anemia can be caused by COVID-19-induced hypoplastic bone marrow and antiviral therapy adverse effects [61]. Another major explanation might be iron insufficiency of vitamin B12 and folate deficit caused by low appetite and anxiety in COVID-19 patients [61]; however, there are scarce data in this critical condition. In this cohort of COVID-19 survivors, all three MICT, RT, and CET treatments caused significant improvements in hemoglobin levels at both 4 and 8 weeks. Increased red blood cell counts and hematocrit concentrations at these time points support this view. Exercise training has been shown to increase total hemoglobin and red blood cell mass, resulting in increased blood oxygen-carrying capacity. The underlying mechanism is thought to originate mostly in the bone marrow and includes enhanced erythropoiesis with hematopoietic bone marrow hyperplasia, improved hematopoietic microenvironment produced by exercise training, and hormone- and cytokine-accelerated erythropoiesis [62].

### Strengths and Limitations of This Study

One concern with the current study's findings was that, for logistical reasons, lack of resources, and the inadequate number of patients in each category, we were unable to do randomization based on gender and disease severity. However, to the best of our knowledge, this is the first randomized controlled trial in which the direct effects of different exercise modalities have been compared before and after training with a control group concerning the biochemical and hematologic markers in patients surviving COVID-19. Post-registration changes in the protocol are another limitation of the current implementation. Because of the topic's significance, in the present study, we decided to look into prognostic hematological and biochemical indicators in blood samples using a smaller population and shorter time frame than the original design. Therefore, to investigate how varied physical activity patterns over eight weeks would affect prognostic blood indicators of the disease, the participants of the present study were randomly chosen from the subjects of the original design. In the present design, there were no subjective measures of COVID-19 symptoms; thus, it is unclear whether these alterations in circulatory biomarkers are linked to subjective symptom reports. Our laboratory is presently addressing these issues. Another possible limitation consists of self-reported data during the study period. Furthermore, due to the nature of the trial, it was impossible to mask the given intervention, but we did blind the outcome assessments, which is all we can be expected to do with this type of study. Moreover, possible confounders such as medication usage were not considered. As a result, the influence of confounding will be a key topic for future research. However, despite these limitations, before this trial, the responses of biochemical and hematologic parameters linked to COVID-19 symptoms and severity to exercise in surviving patients were unclear. Importantly, this research adds weight to the previous findings and goes beyond our prior understandings by demonstrating that COVID-19 survivors benefit from exercise training. More research in bigger cohorts with varied training designs is needed to better understand the effects of exercise modalities on markers associated with symptoms and severity in surviving COVID-19 patients.

Due to the significance and interest of the topic, we decided to look into prognostic hematological and biochemical indicators in blood samples in the current study using a smaller population and shorter timeframe than the initial plan. In order to investigate how varied patterns of physical activity in patients over an eight-week period affected prognostic blood indicators of the illness, the participants of the present study were randomly chosen from the subjects of the primary design.

## Conclusions

Findings from this study demonstrated the efficacy of home-based MICT, RT, and CET interventions in accelerating and enhancing the recovery in patients surviving COVID-19. In detail, the biochemical and hematologic markers linked to the symptoms and severity of COVID-19 improved significantly after eight weeks of MICT, RT, or CET. These findings further indicate that CET was significantly more effective than MICT and RT in improving these variables. The study also demonstrated the feasibility and acceptance of home-based exercise regimens among COVID-19 survivors, which may be utilized to develop upcoming interventions. Given that the trial results were promising, it may be adopted within the medical framework for COVID-19 survivors' treatment and follow-up in primary or secondary care settings. Additional research is demanded to confirm this novel finding.

## Supplementary Information

**Additional file 1.** Effect size and descriptive measures by intervention groups.

## Data Availability

All data are available in the manuscript or the Additional file 1. Additional information would be available by request.

## Abbreviations

ANOVA: Analysis of variance
CET: Combined aerobic and resistance training
CK: Creatine kinase
CRP: C-reactive protein
FFQ: Food frequency questionnaire
HR: Heart rate
K: Potassium
LDH: Lactate dehydrogenase
MICT: Moderate-intensity continuous training
Na: Sodium
NON-EX: Non-exercise
RBC: Red blood cells
RT: Resistance training
WBC: White blood cell
WHO: World Health Organization
