# Supplementary material for: The Effects of Exercise Training on Recovery of Biochemical and Hematological Outcomes in Patients Surviving COVID-19: A Randomized Controlled Assessor-Blinded Trial
Source: Sports Med Open. 2022 Dec 23;8:152. doi: 10.1186/s40798-022-00546-4 (PMC9782268; doi:10.1186/s40798-022-00546-4)
Supplement: Supplementary file 1 — Additional file 1. Effect size and descriptive measures by intervention groups. [file 40798_2022_546_MOESM1_ESM.docx]

Table S1. Mean and standard deviation of the study outcomes within three time measures, and Eta Squared Effect Size of follow-up measurements between the four study interventions

| Variable | NON-EX (n=65) | MICT (n = 47) | RT (n = 28) | CET (n = 72) | Eta Squared Effect Size |
| --- | --- | --- | --- | --- | --- |
| WBC  Time 0  Time 1  Time 2 | 9246.15±5174.70  8123.07±3072.39  7413.85±2290.60 | 7027.66±3692.39  6693.61±2362.15  6163.83^a^±1190.62 | 7221.42±4310.48  6503.57±2897.70  5957.14^a^±1501.71 | 8563.88±4112.31  7613.88±2649.12  6327.77^a^±1046.01 | 0.026  0.186 |
| Neutrophil  Time 0  Time 1  Time 2 | 73.99±13.48  74.85±3.89  71.94±3.29 | 70.02±12.24  68.72^a^±5.53  65.40^a^±6.71 | 68.14±12.40  68.68^a^±5.66  64.87^a^±3.93 | 72.56±14.54  69.76^a^±5.95  61.57^a,b,c^±3.60 | 0.223  0.479 |
| Lymphocytes  Time 0  Time 1  Time 2 | 18.23±11.91  18.59±3.05  21.75±2.71 | 22.19±11.49  22.49^a^±3.04  28.53^a^±3.31 | 23.22±10.30  22.96^a^±4.99  28.49^a^±3.72 | 19.75±12.70  23.21^a^±6.07  31.06^a,b,c^±3.05 | 0.190  0.609 |
| CK  Time 0  Time 1  Time 2 | 121.64±21.83  119.87±15.77  103.15±12.56 | 132.91±20.36  116.29^a^±15.33  87.87^a^±11.40 | 131.85±22.73  114.85^a^±18.98  88.46^a^±16.89 | 129.51±28.29  115.15^a^±11.81  70.63^a,b,c^±19.37 | 0.102  0.506 |
| LDH  Time 0  Time 1  Time 2 | 658.12±354.91  671.20±260.79  542.63±160.96 | 553.40±162.12  536.42^a^±73.26  271.02^a^±50.02 | 524.39±139.96  532.28±90.34  274.75^a^±48.04 | 648.59±305.34  638.95±219.01  278.47^a^±64.29 | 0.070  0.653 |
| CRP  Time 0  Time 1  Time 2 | 27.65±7.13  18.65±4.12  8.78±2.38 | 25.52±4.97  5.30^a^±1.15  1.87^a^±0.98 | 24.26±6.37  5.23^a^±1.06  1.91^a^±0.85 | 26.64±6.59  5.75^a^±1.82  2.21^a^±1.05 | 0.888  0.843 |
| Troponin-I  Time 0  Time 1  Time 2 | 772.54±350.95  281.45±55.87  185.43±28.91 | 627.46±281.27  168.41^a^±17.23  15.38^a^±3.41 | 603.79±198.26  167.38^a^±15.82  13.48^a^±3.20 | 748.71±327.35  177.54^a^±19.69  16.33^a^±4.47 | 0.733  0.966 |
| Platelets  Time 0  Time 1  Time 2 | 225815.38±70240.63  116707.69±8681.37  129984.61±23411.73 | 213361.70±46968.73  145468.08^a^±15732.61  243234.04^a^±43704.64 | 199357.14±40992.51  142821.42a±19516.47  254357.14a±38545.99 | 234152.77±71416.97  153069.44^a^±22966.80  289333.33^a,b,c^±40242.92 | 0.466  0.769 |
| DDIMER  Time 0  Time 1  Time 2 | 1.20±0.63  0.73±0.30  0.68±0.66 | 0.92±0.26  0.44^a^±0.07  0.19^a^±0.06 | 1.04±0.24  0.40^a^±0.06  0.17^a^±0.05 | 1.02±0.86  0.82^b,c^±0.59  0.19^a^±0.06 | 0.170  0.281 |
| RBC  Time 0  Time 1  Time 2 | 4.57±0.61  3.41±0.38  3.88±0.32 | 4.60±0.52  3.93^a^±0.27  4.33^a^±0.21 | 4.60±0.43  3.92^a^±0.14  4.33^a^±0.20 | 4.55±0.59  3.86^a^±0.30  4.39^a^±0.26 | 0.398  0.444 |
| Hemoglobin  Time 0  Time 1  Time 2 | 12.81±2.31  9.37±1.50  11.48±1.37 | 13.80±1.97  11.97^a^±1.14  14.00^a^±0.65 | 13.95±1.75  12.22^a^±0.85  14.36^a^±0.66 | 13.30±1.91  11.72^a^±1.51  14.60^a,b^±0.66 | 0.451  0.697 |
| Hematocrit  Time 0  Time 1  Time 2 | 37.33±6.65  34.05±4.81  36.00±3.75 | 38.36±4.33  37.33^a^±3.09  42.66^a^±2.71 | 40.04±4.31  38.01^a^±1.99  43.69^a^±2.30 | 38.46±5.62  35.93^a,b^±3.99  45.14^a,b,c^±2.69 | 0.209  0.655 |
| K  Time 0  Time 1  Time 2 | 3.72±0.43  4.01±0.16  4.16±0.14 | 3.64±0.34  4.05^a^±0.16  4.72^a^±0.26 | 3.77±0.36  4.14^a^±0.12  4.79^a^±0.21 | 3.74±0.37  4.11±0.18  5.02^a,b,c^±0.15 | 0.105  0.787 |
| NA  Time 0  Time 1  Time 2 | 136.97±5.77  140.21±1.09  141.43±1.10 | 138.14±1.10  140.97^a^±0.73  143.40^a^±0.71 | 138.39±1.22  141.32^a^±0.72  143.71^a^±0.94 | 137.89±1.79  141.19^a^±1.14  144.34^a,b,c^±0.69 | 0.149  0.655 |
| UREA  Time 0  Time 1  Time 2 | 43.69±22.41  40.24±16.11  33.58±12.24 | 33.91±10.00  31.85±5.93  24.25^a^±4.59 | 30.57±5.30  30.64±4.77  23.85^a^±3.87 | 41.62±17.90  38.25±13.53  23.72^a^±4.18 | 0.018  0.286 |
| Creatinine  Time 0  Time 1  Time 2 | 1.15±0.27  1.06±0.17  1.01±0.13 | 1.09±0.26  0.97^a^±0.13  0.81^a^±0.10 | 1.05±0.22  0.94^a^±0.13  0.79^a^±0.08 | 1.12±0.26  0.98^a^±0.13  0.76^a,b^±0.07 | 0.098  0.594 |

MICT = moderate intensity continuous training; RT: resistance training; CET: combined exercise training; NON-EX = non-exercise.

Time0: baseline measurements

Time1: 24 hrs after the last training session in week 4.

Time2: 24 hrs after the last training session in week 8.
